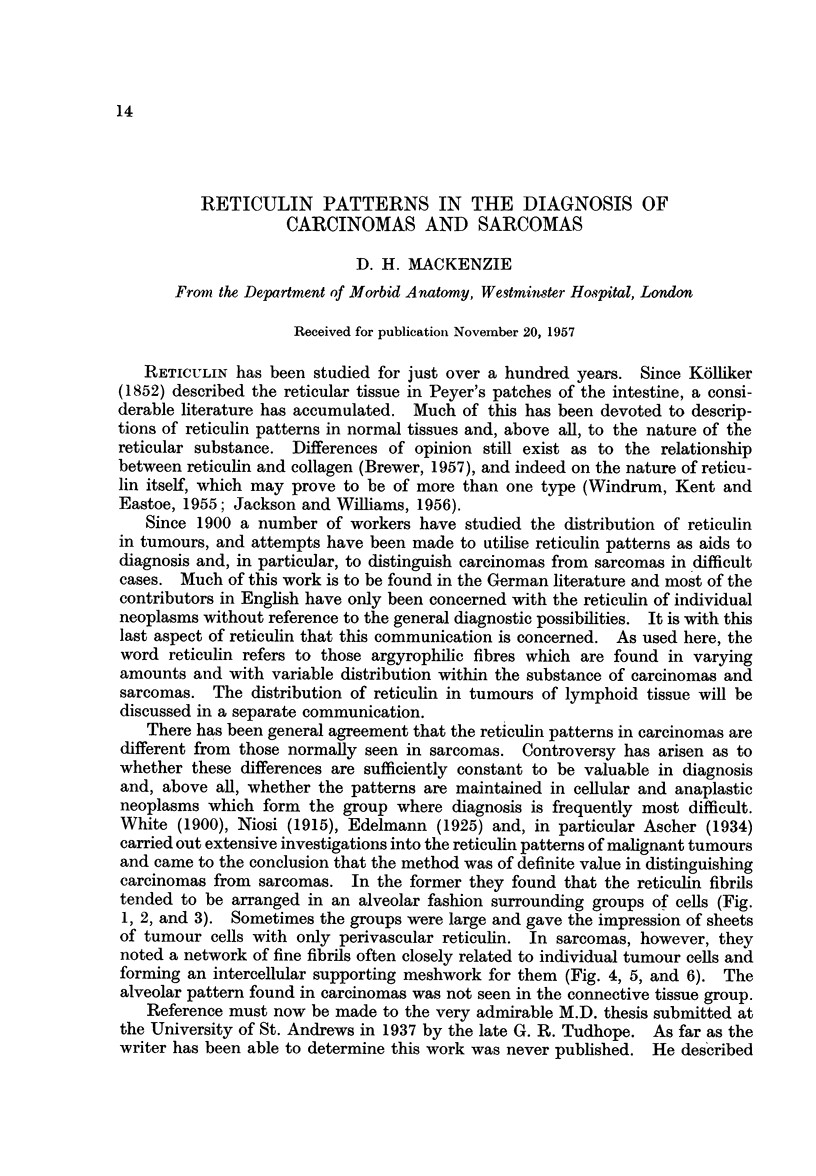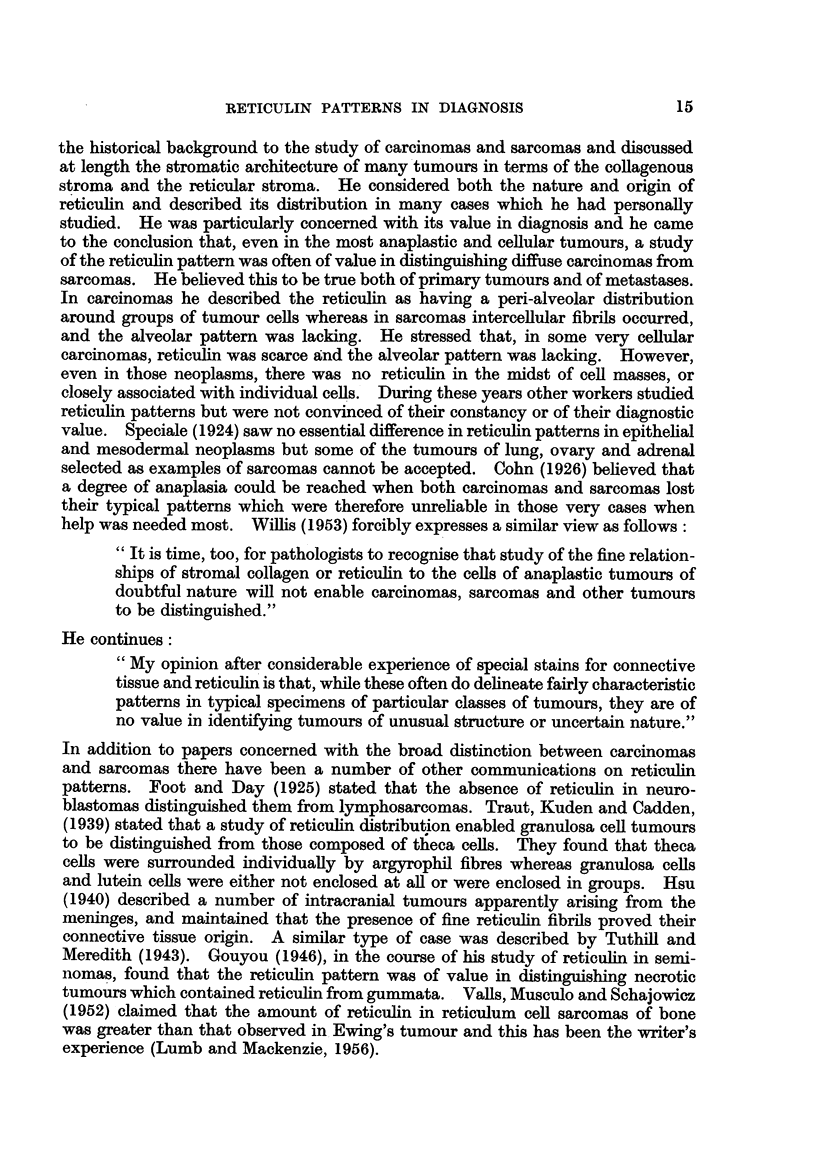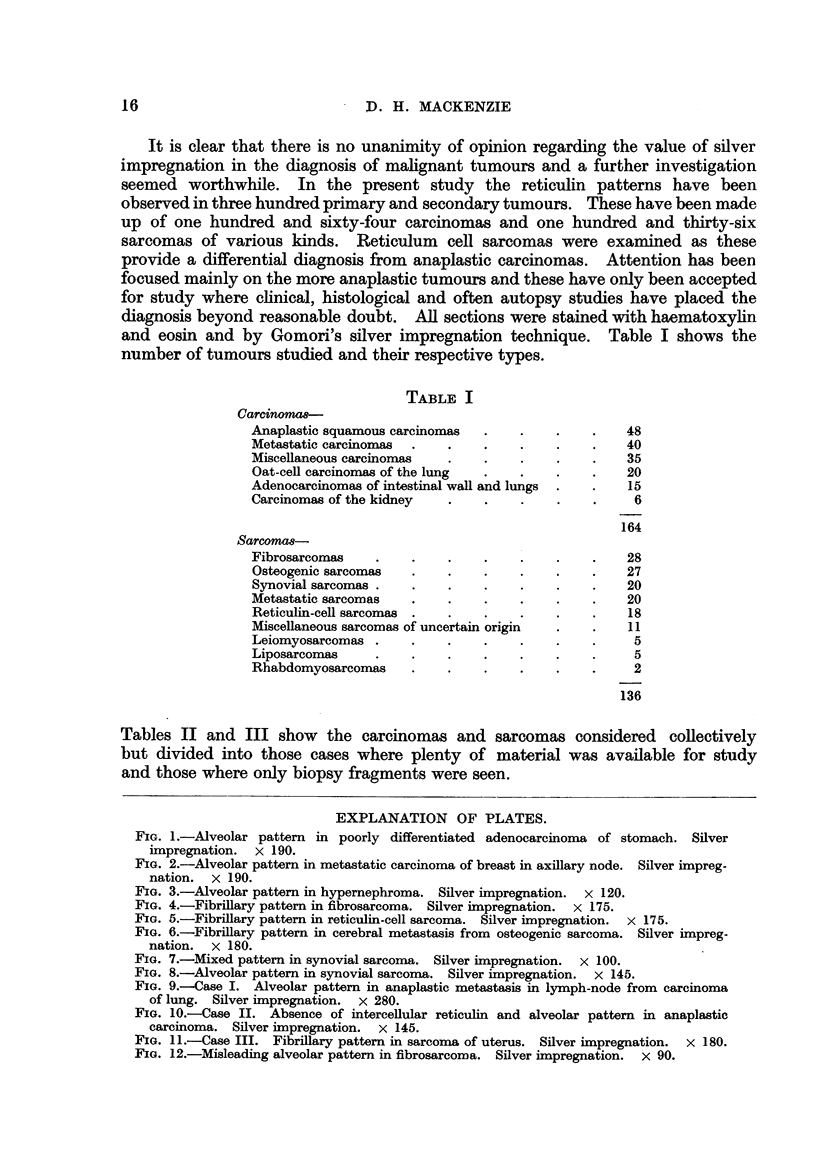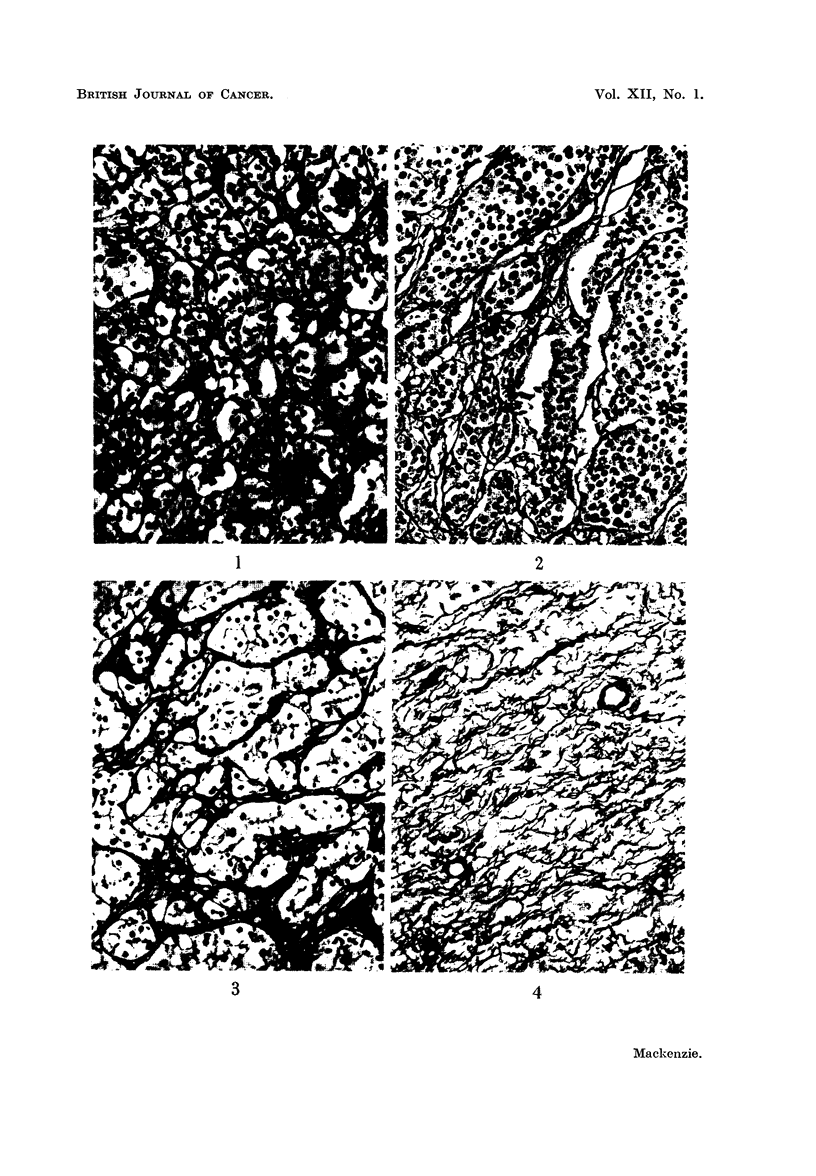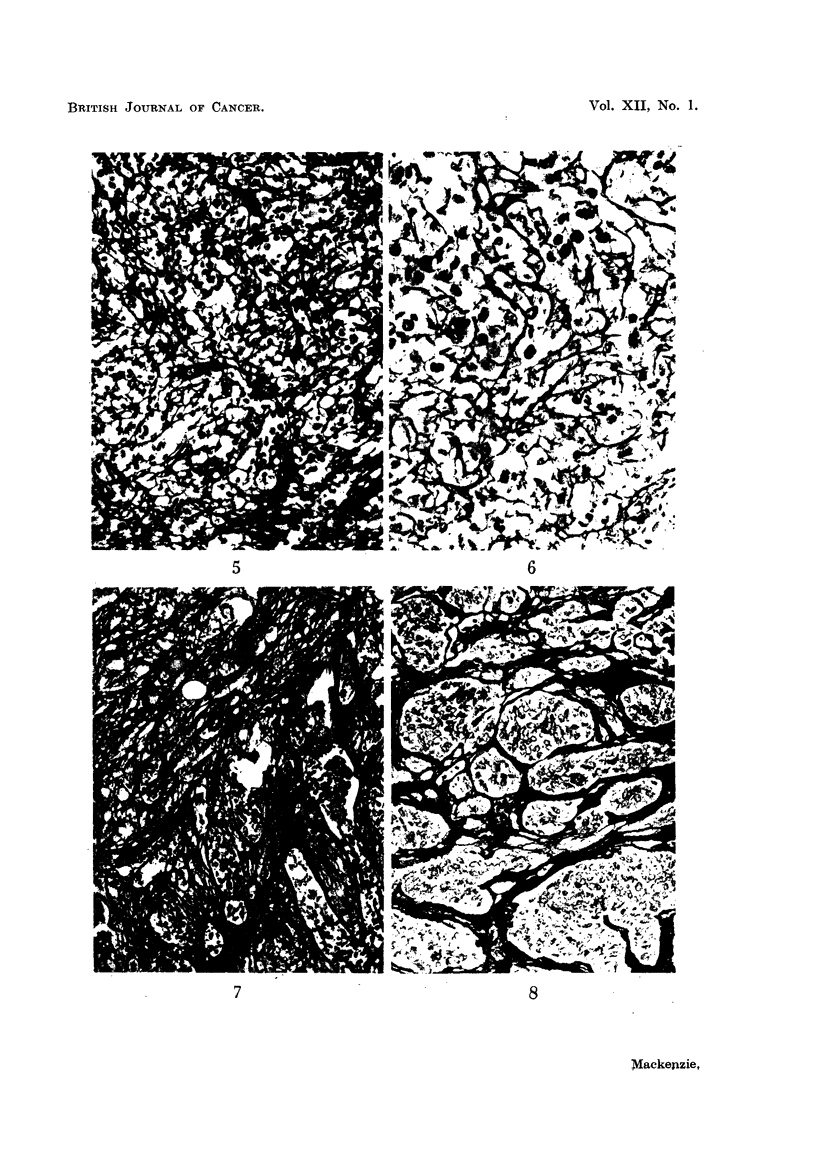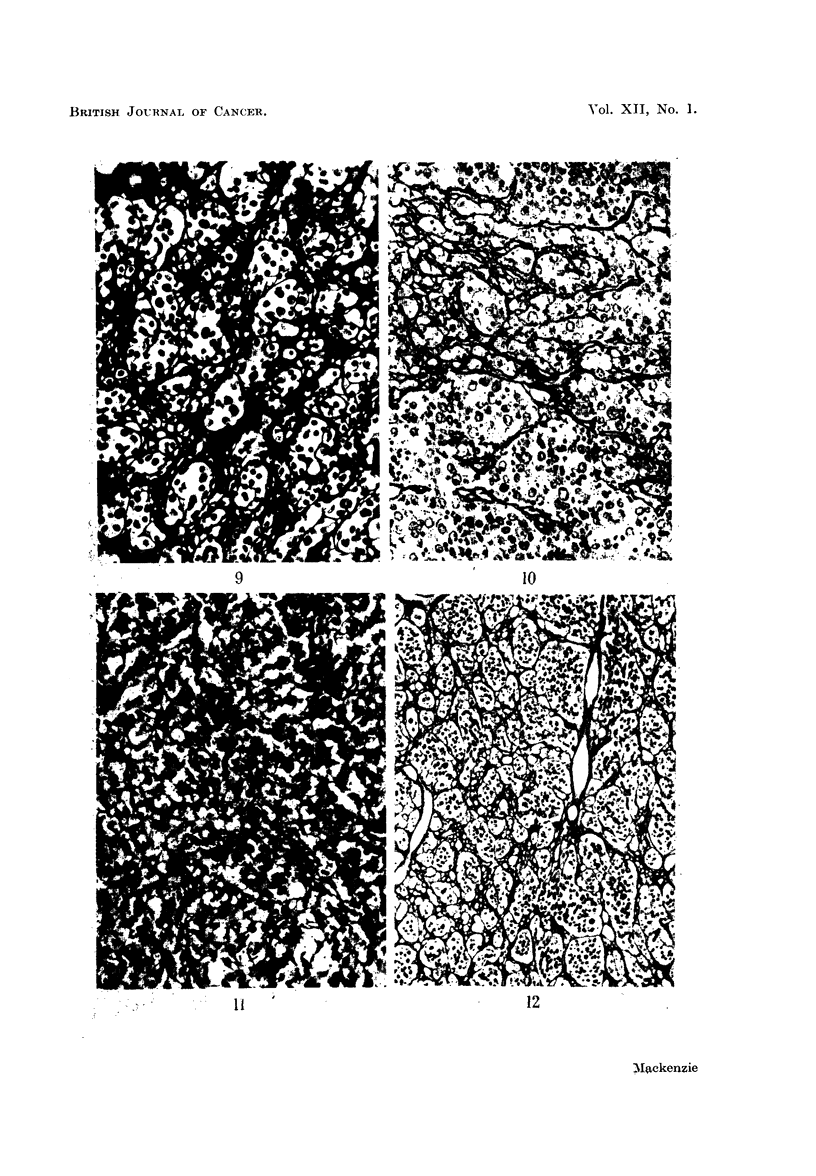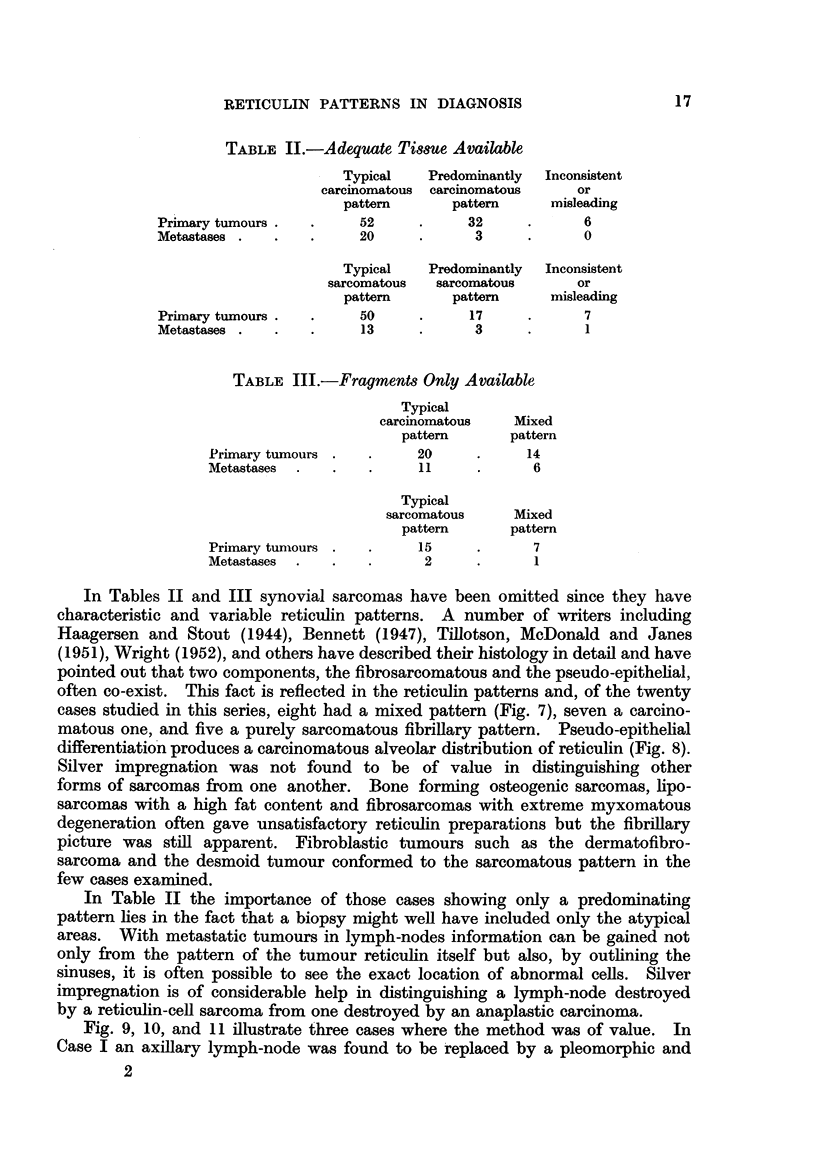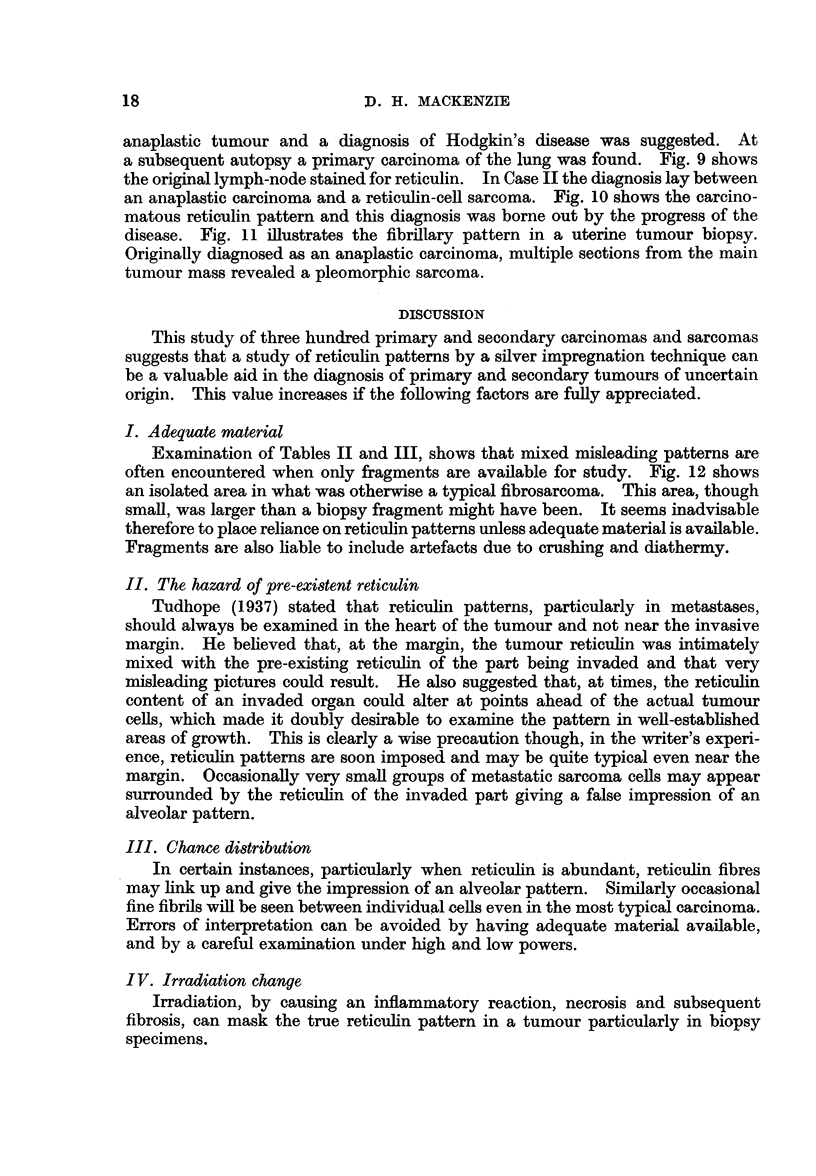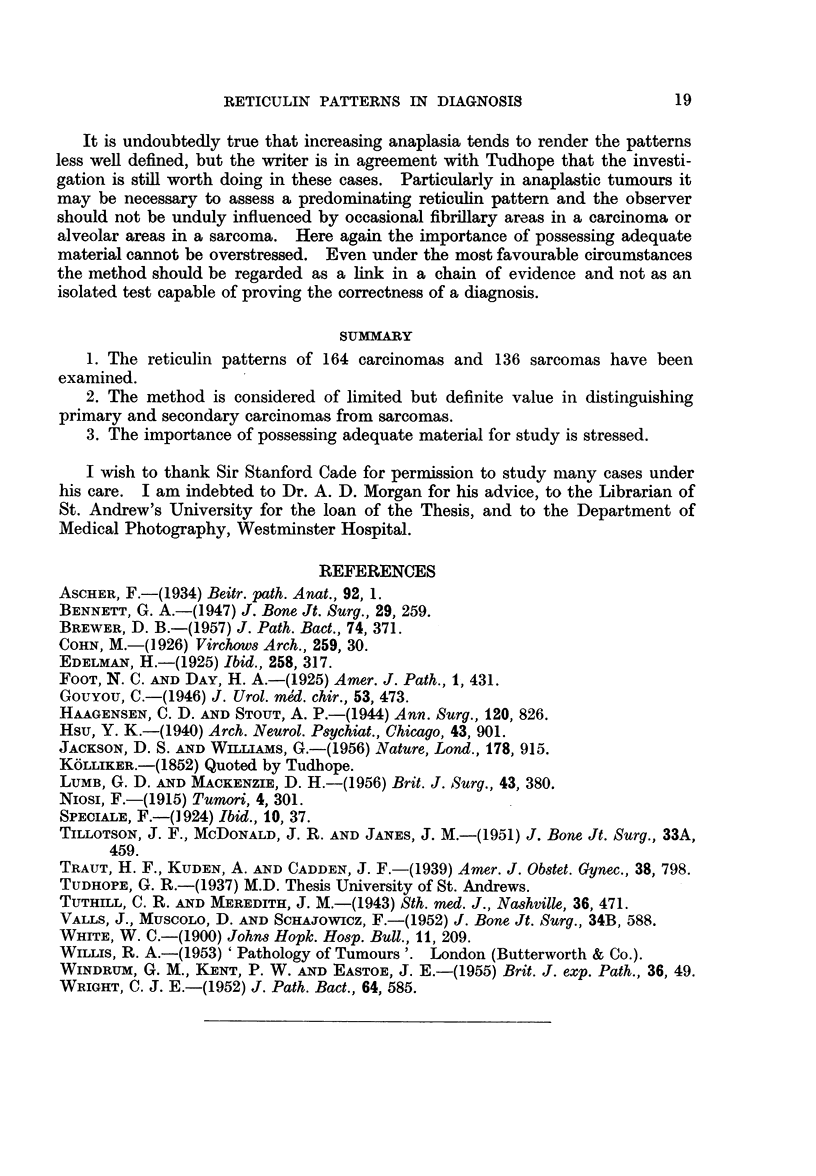# Reticulin Patterns in the Diagnosis of Carcinomas and Sarcomas

**DOI:** 10.1038/bjc.1958.3

**Published:** 1958-03

**Authors:** D. H. Mackenzie

## Abstract

**Images:**


### RETICULIN PATTERNS IN THE DIAGNOSIS OF CARCINOMAS AND SARCOMAS

D. H. MACKENZIE

*From the Department of Morbid Anatomy, Westminster Hospital, London*

Received for publication November 20, 1957

RETICULIN has been studied for just over a hundred years. Since Kölliker (1852) described the reticular tissue in Peyer's patches of the intestine, a considerable literature has accumulated. Much of this has been devoted to descriptions of reticulin patterns in normal tissues and, above all, to the nature of the reticular substance. Differences of opinion still exist as to the relationship between reticulin and collagen (Brewer, 1957), and indeed on the nature of reticulin itself, which may prove to be of more than one type (Windrum, Kent and Eastoe, 1955; Jackson and Williams, 1956).

Since 1900 a number of workers have studied the distribution of reticulin in tumours, and attempts have been made to utilise reticulin patterns as aids to diagnosis and, in particular, to distinguish carcinomas from sarcomas in difficult cases. Much of this work is to be found in the German literature and most of the contributors in English have only been concerned with the reticulin of individual neoplasms without reference to the general diagnostic possibilities. It is with this last aspect of reticulin that this communication is concerned. As used here, the word reticulin refers to those argyrophilic fibres which are found in varying amounts and with variable distribution within the substance of carcinomas and sarcomas. The distribution of reticulin in tumours of lymphoid tissue will be discussed in a separate communication.

There has been general agreement that the reticulin patterns in carcinomas are different from those normally seen in sarcomas. Controversy has arisen as to whether these differences are sufficiently constant to be valuable in diagnosis and, above all, whether the patterns are maintained in cellular and anaplastic neoplasms which form the group where diagnosis is frequently most difficult. White (1900), Niosi (1915), Edelmann (1925) and, in particular Ascher (1934) carried out extensive investigations into the reticulin patterns of malignant tumours and came to the conclusion that the method was of definite value in distinguishing carcinomas from sarcomas. In the former they found that the reticulin fibrils tended to be arranged in an alveolar fashion surrounding groups of cells (Fig. 1, 2, and 3). Sometimes the groups were large and gave the impression of sheets of tumour cells with only perivascular reticulin. In sarcomas, however, they noted a network of fine fibrils often closely related to individual tumour cells and forming an intercellular supporting meshwork for them (Fig. 4, 5, and 6). The alveolar pattern found in carcinomas was not seen in the connective tissue group.

Reference must now be made to the very admirable M.D. thesis submitted at the University of St. Andrews in 1937 by the late G. R. Tudhope. As far as the writer has been able to determine this work was never published. He described the historical background to the study of carcinomas and sarcomas and discussed at length the stromatic architecture of many tumours in terms of the collagenous stroma and the reticular stroma. He considered both the nature and origin of reticulin and described its distribution in many cases which he had personally studied. He was particularly concerned with its value in diagnosis and he came to the conclusion that, even in the most anaplastic and cellular tumours, a study of the reticulin pattern was often of value in distinguishing diffuse carcinomas from sarcomas. He believed this to be true both of primary tumours and of metastases. In carcinomas he described the reticulin as having a peri-alveolar distribution around groups of tumour cells whereas in sarcomas intercellular fibrils occurred, and the alveolar pattern was lacking. He stressed that, in some very cellular carcinomas, reticulin was scarce and the alveolar pattern was lacking. However, even in those neoplasms, there was no reticulin in the midst of cell masses, or closely associated with individual cells. During these years other workers studied reticulin patterns but were not convinced of their constancy or of their diagnostic value. Speciale (1924) saw no essential difference in reticulin patterns in epithelial and mesodermal neoplasms but some of the tumours of lung, ovary and adrenal selected as examples of sarcomas cannot be accepted. Cohn (1926) believed that a degree of anaplasia could be reached when both carcinomas and sarcomas lost their typical patterns which were therefore unreliable in those very cases when help was needed most. Willis (1953) forcibly expresses a similar view as follows :

> " It is time, too, for pathologists to recognise that study of the fine relationships of stromal collagen or reticulin to the cells of anaplastic tumours of doubtful nature will not enable carcinomas, sarcomas and other tumours to be distinguished."

He continues :

> " My opinion after considerable experience of special stains for connective tissue and reticulin is that, while these often do delineate fairly characteristic patterns in typical specimens of particular classes of tumours, they are of no value in identifying tumours of unusual structure or uncertain nature."

In addition to papers concerned with the broad distinction between carcinomas and sarcomas there have been a number of other communications on reticulin patterns. Foot and Day (1925) stated that the absence of reticulin in neuroblastomas distinguished them from lymphosarcomas. Traut, Kuden and Cadden, (1939) stated that a study of reticulin distribution enabled granulosa cell tumours to be distinguished from those composed of theca cells. They found that theca cells were surrounded individually by argyrophil fibres whereas granulosa cells and lutein cells were either not enclosed at all or were enclosed in groups. Hsu (1940) described a number of intracranial tumours apparently arising from the meninges, and maintained that the presence of fine reticulin fibrils proved their connective tissue origin. A similar type of case was described by Tuthill and Meredith (1943). Gouyou (1946), in the course of his study of reticulin in seminomas, found that the reticulin pattern was of value in distinguishing necrotic tumours which contained reticulin from gummata. Valls, Musculo and Schajowicz (1952) claimed that the amount of reticulin in reticulum cell sarcomas of bone was greater than that observed in Ewing's tumour and this has been the writer's experience (Lumb and Mackenzie, 1956).

It is clear that there is no unanimity of opinion regarding the value of silver impregnation in the diagnosis of malignant tumours and a further investigation seemed worthwhile. In the present study the reticulin patterns have been observed in three hundred primary and secondary tumours. These have been made up of one hundred and sixty-four carcinomas and one hundred and thirty-six sarcomas of various kinds. Reticulum cell sarcomas were examined as these provide a differential diagnosis from anaplastic carcinomas. Attention has been focused mainly on the more anaplastic tumours and these have only been accepted for study where clinical, histological and often autopsy studies have placed the diagnosis beyond reasonable doubt. All sections were stained with haematoxylin and eosin and by Gomori's silver impregnation technique. Table I shows the number of tumours studied and their respective types.

TABLE I

| | |
|---|---|
| *Carcinomas—* | |
| Anaplastic squamous carcinomas | 48 |
| Metastatic carcinomas | 40 |
| Miscellaneous carcinomas | 35 |
| Oat-cell carcinomas of the lung | 20 |
| Adenocarcinomas of intestinal wall and lungs | 15 |
| Carcinomas of the kidney | 6 |
| | 164 |
| *Sarcomas—* | |
| Fibrosarcomas | 28 |
| Osteogenic sarcomas | 27 |
| Synovial sarcomas | 20 |
| Metastatic sarcomas | 20 |
| Reticulin-cell sarcomas | 18 |
| Miscellaneous sarcomas of uncertain origin | 11 |
| Leiomyosarcomas | 5 |
| Liposarcomas | 5 |
| Rhabdomyosarcomas | 2 |
| | 136 |

Tables II and III show the carcinomas and sarcomas considered collectively but divided into those cases where plenty of material was available for study and those where only biopsy fragments were seen.

EXPLANATION OF PLATES.

FIG. 1.—Alveolar pattern in poorly differentiated adenocarcinoma of stomach. Silver impregnation. × 190.

FIG. 2.—Alveolar pattern in metastatic carcinoma of breast in axillary node. Silver impregnation. × 190.

FIG. 3.—Alveolar pattern in hypernephroma. Silver impregnation. × 120.

FIG. 4.—Fibrillary pattern in fibrosarcoma. Silver impregnation. × 175.

FIG. 5.—Fibrillary pattern in reticulin-cell sarcoma. Silver impregnation. × 175.

FIG. 6.—Fibrillary pattern in cerebral metastasis from osteogenic sarcoma. Silver impregnation. × 180.

FIG. 7.—Mixed pattern in synovial sarcoma. Silver impregnation. × 100.

FIG. 8.—Alveolar pattern in synovial sarcoma. Silver impregnation. × 145.

FIG. 9.—Case I. Alveolar pattern in anaplastic metastasis in lymph-node from carcinoma of lung. Silver impregnation. × 280.

FIG. 10.—Case II. Absence of intercellular reticulin and alveolar pattern in anaplastic carcinoma. Silver impregnation. × 145.

FIG. 11.—Case III. Fibrillary pattern in sarcoma of uterus. Silver impregnation. × 180.

FIG. 12.—Misleading alveolar pattern in fibrosarcoma. Silver impregnation. × 90.

1

2

3

4

5 6 7 8

9

10

11

12

Mackenzie

TABLE II.—*Adequate Tissue Available*

| | Typical carcinomatous pattern | Predominantly carcinomatous pattern | Inconsistent or misleading |
|---|---|---|---|
| Primary tumours | 52 | 32 | 6 |
| Metastases | 20 | 3 | 0 |

| | Typical sarcomatous pattern | Predominantly sarcomatous pattern | Inconsistent or misleading |
|---|---|---|---|
| Primary tumours | 50 | 17 | 7 |
| Metastases | 13 | 3 | 1 |

TABLE III.—*Fragments Only Available*

| | Typical carcinomatous pattern | Mixed pattern |
|---|---|---|
| Primary tumours | 20 | 14 |
| Metastases | 11 | 6 |

| | Typical sarcomatous pattern | Mixed pattern |
|---|---|---|
| Primary tumours | 15 | 7 |
| Metastases | 2 | 1 |

In Tables II and III synovial sarcomas have been omitted since they have characteristic and variable reticulin patterns. A number of writers including Haagersen and Stout (1944), Bennett (1947), Tillotson, McDonald and Janes (1951), Wright (1952), and others have described their histology in detail and have pointed out that two components, the fibrosarcomatous and the pseudo-epithelial, often co-exist. This fact is reflected in the reticulin patterns and, of the twenty cases studied in this series, eight had a mixed pattern (Fig. 7), seven a carcinomatous one, and five a purely sarcomatous fibrillary pattern. Pseudo-epithelial differentiation produces a carcinomatous alveolar distribution of reticulin (Fig. 8). Silver impregnation was not found to be of value in distinguishing other forms of sarcomas from one another. Bone forming osteogenic sarcomas, liposarcomas with a high fat content and fibrosarcomas with extreme myxomatous degeneration often gave unsatisfactory reticulin preparations but the fibrillary picture was still apparent. Fibroblastic tumours such as the dermatofibrosarcoma and the desmoid tumour conformed to the sarcomatous pattern in the few cases examined.

In Table II the importance of those cases showing only a predominating pattern lies in the fact that a biopsy might well have included only the atypical areas. With metastatic tumours in lymph-nodes information can be gained not only from the pattern of the tumour reticulin itself but also, by outlining the sinuses, it is often possible to see the exact location of abnormal cells. Silver impregnation is of considerable help in distinguishing a lymph-node destroyed by a reticulin-cell sarcoma from one destroyed by an anaplastic carcinoma.

Fig. 9, 10, and 11 illustrate three cases where the method was of value. In Case I an axillary lymph-node was found to be replaced by a pleomorphic and anaplastic tumour and a diagnosis of Hodgkin's disease was suggested. At a subsequent autopsy a primary carcinoma of the lung was found. Fig. 9 shows the original lymph-node stained for reticulin. In Case II the diagnosis lay between an anaplastic carcinoma and a reticulin-cell sarcoma. Fig. 10 shows the carcinomatous reticulin pattern and this diagnosis was borne out by the progress of the disease. Fig. 11 illustrates the fibrillary pattern in a uterine tumour biopsy. Originally diagnosed as an anaplastic carcinoma, multiple sections from the main tumour mass revealed a pleomorphic sarcoma.

### DISCUSSION

This study of three hundred primary and secondary carcinomas and sarcomas suggests that a study of reticulin patterns by a silver impregnation technique can be a valuable aid in the diagnosis of primary and secondary tumours of uncertain origin. This value increases if the following factors are fully appreciated.

### *I. Adequate material*

Examination of Tables II and III, shows that mixed misleading patterns are often encountered when only fragments are available for study. Fig. 12 shows an isolated area in what was otherwise a typical fibrosarcoma. This area, though small, was larger than a biopsy fragment might have been. It seems inadvisable therefore to place reliance on reticulin patterns unless adequate material is available. Fragments are also liable to include artefacts due to crushing and diathermy.

### *II. The hazard of pre-existent reticulin*

Tudhope (1937) stated that reticulin patterns, particularly in metastases, should always be examined in the heart of the tumour and not near the invasive margin. He believed that, at the margin, the tumour reticulin was intimately mixed with the pre-existing reticulin of the part being invaded and that very misleading pictures could result. He also suggested that, at times, the reticulin content of an invaded organ could alter at points ahead of the actual tumour cells, which made it doubly desirable to examine the pattern in well-established areas of growth. This is clearly a wise precaution though, in the writer's experience, reticulin patterns are soon imposed and may be quite typical even near the margin. Occasionally very small groups of metastatic sarcoma cells may appear surrounded by the reticulin of the invaded part giving a false impression of an alveolar pattern.

### *III. Chance distribution*

In certain instances, particularly when reticulin is abundant, reticulin fibres may link up and give the impression of an alveolar pattern. Similarly occasional fine fibrils will be seen between individual cells even in the most typical carcinoma. Errors of interpretation can be avoided by having adequate material available, and by a careful examination under high and low powers.

### *IV. Irradiation change*

Irradiation, by causing an inflammatory reaction, necrosis and subsequent fibrosis, can mask the true reticulin pattern in a tumour particularly in biopsy specimens.

It is undoubtedly true that increasing anaplasia tends to render the patterns less well defined, but the writer is in agreement with Tudhope that the investigation is still worth doing in these cases. Particularly in anaplastic tumours it may be necessary to assess a predominating reticulin pattern and the observer should not be unduly influenced by occasional fibrillary areas in a carcinoma or alveolar areas in a sarcoma. Here again the importance of possessing adequate material cannot be overstressed. Even under the most favourable circumstances the method should be regarded as a link in a chain of evidence and not as an isolated test capable of proving the correctness of a diagnosis.

### SUMMARY

1. The reticulin patterns of 164 carcinomas and 136 sarcomas have been examined.

2. The method is considered of limited but definite value in distinguishing primary and secondary carcinomas from sarcomas.

3. The importance of possessing adequate material for study is stressed.

I wish to thank Sir Stanford Cade for permission to study many cases under his care. I am indebted to Dr. A. D. Morgan for his advice, to the Librarian of St. Andrew's University for the loan of the Thesis, and to the Department of Medical Photography, Westminster Hospital.

### REFERENCES

Ascher, F.—(1934) *Beitr. path. Anat.*, **92**, 1.
Bennett, G. A.—(1947) *J. Bone Jt. Surg.*, **29**, 259.
Brewer, D. B.—(1957) *J. Path. Bact.*, **74**, 371.
Cohn, M.—(1926) *Virchows Arch.*, **259**, 30.
Edelman, H.—(1925) *Ibid.*, **258**, 317.
Foot, N. C. and Day, H. A.—(1925) *Amer. J. Path.*, **1**, 431.
Gouyou, C.—(1946) *J. Urol. méd. chir.*, **53**, 473.
Haagensen, C. D. and Stout, A. P.—(1944) *Ann. Surg.*, **120**, 826.
Hsu, Y. K.—(1940) *Arch. Neurol. Psychiat., Chicago*, **43**, 901.
Jackson, D. S. and Williams, G.—(1956) *Nature, Lond.*, **178**, 915.
Kölliker.—(1852) Quoted by Tudhope.
Lumb, G. D. and Mackenzie, D. H.—(1956) *Brit. J. Surg.*, **43**, 380.
Niosi, F.—(1915) *Tumori*, **4**, 301.
Speciale, F.—(1924) *Ibid.*, **10**, 37.
Tillotson, J. F., McDonald, J. R. and Janes, J. M.—(1951) *J. Bone Jt. Surg.*, **33A**, 459.
Traut, H. F., Kuden, A. and Cadden, J. F.—(1939) *Amer. J. Obstet. Gynec.*, **38**, 798.
Tudhope, G. R.—(1937) M.D. Thesis University of St. Andrews.
Tuthill, C. R. and Meredith, J. M.—(1943) *Sth. med. J., Nashville*, **36**, 471.
Valls, J., Muscolo, D. and Schajowicz, F.—(1952) *J. Bone Jt. Surg.*, **34B**, 588.
White, W. C.—(1900) *Johns Hopk. Hosp. Bull.*, **11**, 209.
Willis, R. A.—(1953) ' Pathology of Tumours '. London (Butterworth & Co.).
Windrum, G. M., Kent, P. W. and Eastoe, J. E.—(1955) *Brit. J. exp. Path.*, **36**, 49.
Wright, C. J. E.—(1952) *J. Path. Bact.*, **64**, 585.